# Towards a better understanding of attitudes and beliefs held by traditional healers and recipients of traditional medicine concerning mental health conditions in post-conflict Liberia: a qualitative investigation

**DOI:** 10.4314/ahs.v21i3.51

**Published:** 2021-09

**Authors:** Samuel J Pullen, Augusta R Herman, Brittany CL Lange, Nicole Christian-Brathwaite, Melissa Ulloa, Michael P Kempeh, Dyujay G Karnga, Mosoka P Fallah, Jeremiah Menyongai, Benjamin Harris, Yadira Alonso, David C Henderson, Christina PC Borba

**Affiliations:** 1 Duke University School of Medicine, Department of Psychiatry and Behavioral Sciences, Division of Child and Family Mental Health and Community Psychiatry, Durham, NC; 2 Johns Hopkins Bloomberg School of Public Health, Baltimore; 3 Public Health at Seattle King County, Seattle, WA; 4 University of Oxford – Department of Social Policy and Intervention, Oxford, England; 5 Well Minds Consulting Company, 95 Eastern Ave, Dedham, MA 02026; 6 New York University Steinhardt School of Culture, Education, and Human Development, 82 Washington Square E, New York, NY 10003; 7 A.M. Dogliotti College of Medicine, University of Liberia, Monrovia, Liberia, College of Medicine University of Liberia, Monrovia, Liberia, West Africa; 8 National Public Health Institute of Liberia, Monrovia, Liberia; 9 Christ Jubilee International Ministries, Lowell, MA; 10 Boston University School of Medicine, Department of Psychiatry, Boston Medical Center, Boston, MA

**Keywords:** Traditional healer, mental healthcare, Liberia, qualitative research

## Abstract

**Background:**

A better understanding of attitudes and beliefs held by traditional healers and utilizers of traditional medicine concerning mental health conditions in Liberia is important as Liberia seeks to improve its delivery of mental healthcare in the context of scarce resources and recovery from civil war.

**Methods:**

A qualitative research design was used to collect data from 24 Liberian traditional healers, and 11 utilizers of Liberian traditional medicine. Participants were queried about mental health problems in Liberia, treatments, and attitudes towards modern healthcare. Qualitative data were probed and aggregated using content analysis.

**Results:**

Mental health problems described by study participants included: Open Mole, African Science, Epilepsy, Depression and Mental Illness (trauma/substance use). Mental health problems were often associated with socioeconomic distress, and participants described their attitudes and beliefs concerning mental healthcare, traditional medicine, and modern healthcare.

**Conclusion:**

Traditional medicine is an important part of mental healthcare in Africa. Mental illness, social factors, and healthcare access were important problems in Liberia. Mental health problems blended local cultural beliefs with Westernized nosology and social factors. Traditional healer's attitudes towards Western medicine reflected ambivalence. There is a desire for collaboration with ‘modern’ health care providers, but this will require reciprocal trust-building.

## Introduction

The West African country of Liberia is one of the poorest nations globally and has been plagued by two civil wars spanning almost 15 years^1^,^2^. Communicable and non-communicable diseases are widespread and synergistically strain Liberia's national health system and socioeconomic infrastructure, hindering Liberia's recovery efforts^2^. As an example, the 2014–2016 Ebola outbreak exposed Liberia's inadequate healthcare resources, sowing doubt in the ability of Liberia's health system to respond to the country's many other healthcare challenges, including the significant shortage of resources for Liberia's population suffering from mental illness and substance use disorders (SUD)^3^,^4^,^5^,^6^. The dearth of mental health clinicians have overwhelmed and demoralized the few mental health professionals available to serve Liberia's population and this has further exacerbated the burden of Liberia's national health system to provide adequate mental health services for its population^1^,^2^,^5^,^6^.

To advance mental health system reform, Liberia's government had developed a National Mental Health Policy, most recently revised in 2009^7^,^8^. Consistent with recommendations from the World Health Organizations (WHO) Mental Health Gap Action Program (mhGAP) and the emerging mental health systems in low- and middle-income countries (LMIC) (Emerald) research consortium, Liberia's mental health policy calls for a shift of resources from inpatient psychiatric facilities to community-based mental health facilities, and integration of mental health services into primary care settings and community platforms similar to strategies being developed in other LMIC^7^,^8^,^9^,^10^.

Key contextual factors that drove Liberia's pursuit of mental health system reform included poor access to mental health resources, mental health illiteracy, local customs and cultural beliefs about mental illness, historical mistreatment of mentally ill patients, and fear of Western bio-psychosocial models of care^1^,^2^. Additionally, Liberia's psychiatric hospitals suffer from overcrowding and understaffing, often leading to poor quality of care, and most of Liberia's primary care providers lack training to provide basic mental health care, while Liberia presently only retains two fully trained psychiatrists^1^,^2^. Collectively, such factors often lead Liberians to seek mental health care from informal providers such as community health workers, traditional healers, and religious leaders^1^,^2^. As new generations of Liberians contend with the mounting challenges posed by mental health and substance use disorders, these contextual factors will continue to inform and shape evolving approaches to mental healthcare in Liberia and other developing countries with similar demographic and socioeconomic profiles^11^,^12^.

Westernized approaches to mental healthcare offer a modernized ‘outsiders’ vantage point that may struggle to account for cultural variance in non-western populations, overlooking persons who suffer from cultural idioms of distress that do not conform to predetermined diagnostic criteria^13^. Additionally, attempts to implement modernized approaches to mental healthcare absent local cultural context may also contribute to fear and mistrust of such treatments by the local population^1^,^2^. Thus, a singular focus on etic approaches may fail to fully appreciate cultural context and understanding of mental illness in developing countries^11^,^14^. Therefore, it is important to understand and respect non-western approaches to treatment of mental illness and consider non-western approaches to mental health^11^,^14^.

Traditional healers continue to play an important part in many healthcare delivery models worldwide and are often woven into the fabric of those cultural belief systems, frequently acting as the point of first contact for persons with mental illness^15^,^16^,^17^. Evidence supports a therapeutic and supportive role for traditional healers in providing psychosocial interventions for mental illnesses such as depression or anxiety^17^,^18^,^19^. However, concerns remain about how to safely and effectively integrate traditional healers into primary care and modern healthcare settings^16^,^17^,^18^. Traditional healing typically incorporates emic approaches to healthcare often relying on herbal, mineral, and spiritual-based treatments derived from a summation of knowledge, skills, and practices unique to an indigenous cultural belief system to prevent, assess, and treat persons suffering from somatic or mental illnesses^18^.

As traditional healers continue to be leveraged to provide mental health care in developing countries, this raises a broader contextual question as to how traditional healers best fit within ever-changing healthcare systems that are increasingly relying upon more patient-centered, holistic approaches to integrating mental and physical health care^14^,^20^. Liberia's evolving healthcare paradigm provided an opportunity to further study explanatory models of mental illnesses; from the perspective of the Liberian traditional healer and those they treat^11^,^13^,^14^. Other studies have also investigated the role of traditional healers and utilizers of traditional medicine within health care^17^,^18^. The strength of this study lies in its use of a qualitative research design to solicit a richer, more in-depth understanding of traditional medicine from the perspective of Liberian traditional healers and utilizers of Liberian traditional medicine.

The goals of this study were to gain a better understanding of mental illnesses and treatments used to treat mental illness, social determinants of health in Liberia, and cultural idioms of distress from the perspective of traditional healers and utilizers of traditional medicine. We also wanted to better understand attitudes and beliefs about Western medicine, and perceived role within healthcare from the perspective of traditional healers and utilizers of traditional medicine. By contributing to a more in-depth understanding of the attitudes and beliefs about mental health conditions held by traditional healers and utilizers of traditional medicine our hope is to inform how traditional healers would best fit within a conceptual framework that aims to improve mental healthcare services in post-conflict Liberia, and other developing countries with similar characteristics.

## Methods

### Study Design and Population

A semi-structured, qualitative research study design using purposive-sampling was used to collect information from 24 Liberian “traditional healers” or “herbalists” and 11 Liberians who were treated by a traditional healer. Study participants were recruited from local communities, and traditional medicine clinics in and around Monsterrado County in the Greater Monrovia area. A medical student from the A.M. Dogliotti College of Medicine, University of Liberia was assigned the role as a research assistant and served both as a moderator and translator (henceforth referred to as ‘moderator’) and helped facilitate interviews along with a designated U.S.- based interviewer to obtain data on participant attitudes, beliefs, vocabulary, and thought patterns from each study participant.

Data collection and analysis adhered to the principle of saturation – the point where no new information was likely to be obtained by adding additional interview content^21^,^22^. We attempted to reduce participant bias by using a moderator and interviewer who were not previously known to the participants and were more likely to be emotionally detached from the subject matter in question.

Eligible study participants could be male or female and were required to be able to provide written informed consent. Potential study participants were informed that interviews would last approximately 90 minutes that they were audio-recorded, notes would be taken, and that the moderator may help translate portions of the interview dialogue. Study participants were included if they identified themselves as a traditional healer, and/or if they had ever used traditional medicine in their lifetime. Refreshments or a $5 phone card was provided during the interview as compensation for participation and time taken.

### Data collection

Data was collected from study participants from March 2012 through April 2012. Ethical approval for this study was obtained from the University of Liberia Institutional Review Board, the Partners Healthcare Institutional Review Board, and the Integrated Network for Subject Protection in Research – Boston University Medical Campus Institutional Review Board. Written informed consent was obtained from all study participants. Participants were explicitly given the right to refuse participation or to stop participation at any time. Confidentiality was maintained using study IDs for each participant and all identifying information was removed prior to transcription. Access to study data was restricted to the investigator and the research team. All electronic versions of the documents required password protection and storage on a secure network.

All discussions were held using the preferred language (either Liberian English or Standard English) of the study participant, and if participants were non-English speaking, the moderator helped to facilitate translation of the interview. All interviews were digitally recorded and transcribed verbatim. An expert in qualitative interviewing and coding trained all study staff. Interviews with study participants were recorded and notes were taken by both the primary interviewer and the moderator during and after the interview to ensure accuracy.

### Study measures

Study participants were initially asked open-ended questions inquiring about potential themes such as common problems people in Liberia are dealing with, problem attributions, treatments rendered/received, and under what conditions they would choose to seek care from a traditional healer. Interview questions were guided by a thorough literature review and feedback from the Liberian psychiatrist to inform the content and structure of the questions. Examples of questions included: “What are the main problems that you think people are dealing with in Liberia?”; “What are the main reasons that people seek treatment from you?”; “What do you think the causes of open mole are?” Interviewers then used probing techniques to capture supplemental information regarding the participants' responses to the research questions and to further engage study participants to share their personal experiences.

Traditional healers were asked about the following topics: common healthcare and psychosocial problems in Liberia, attitudes and beliefs towards Western medicine, problem attributions, and modalities commonly used to treat problems they routinely encountered. Likewise, utilizers of traditional medicine were also asked about the following topics: common healthcare and psychosocial problems in Liberia, common reasons that they would see a traditional healer, problem attributions, and treatments rendered by a traditional healer.

### Data analysis

Thematic content analysis was conducted, and data were coded based on reoccurring subject themes and patterns^21^,^22^. After the transcription of interviews with study participants, coders who had undergone formal training in qualitative research, independently read the participant interviews and met to discuss initial perceptions of the transcripts. To preserve the fidelity of the coding process, two members from our research group were required to code the transcription narratives. Upon independently coding the participant interviews, coders met to discuss and agree upon codes to create a preliminary codebook, which was used as a template to identify similar themes when coding subsequent transcription narratives. After completion of the initial codebook, coders independently coded one transcript narrative at a time, and then met for a period lasting 45 minutes up to two hours every other week, for a total of 5 meetings to discuss 1–2 transcript narratives per meeting that were randomly selected by a third member of the research team and agree upon codes and add new codes to the codebook as necessary. A total of 7/35 (20%) transcript narratives were reviewed by both coders in tandem to ensure fidelity of the coding process.

If there were any disagreements among coders, they were managed by discussing the differing perspectives as a dyad and arriving at a consensus. If the coders could not come to a consensus at the time of discussion, the transcript excerpt in question would be recorded on a memo and saved for future discussion after additional transcript narratives were coded to determine if there were recurring themes necessitating a new code be created, or how the transcript excerpt in question would best fit with already established codes.

As coding proceeded, categories were clustered together, from a broad theme of “mental health conditions” to increasingly specific subthemes, such as “open mole”, or “African Science”. A corresponding definition was also created for each code to ensure coding consistency. For each code added to the codebook, a consensus was reached between the coders to ensure inter-coder reliability. NVIVO software was used as a data management tool.

## Results

We interviewed 35 study participants from traditional medicine clinics located in the greater Monrovia area. Study participants were defined as either a traditional healer (self-identified as either a bone specialist, herbalist, healer, or technician) or a utilizer of traditional medicine, and consisted of 15 male traditional healers, 9 female traditional healers, 6 male utilizers of traditional medicine, and 5 female utilizers of traditional medicine [TABLE 1]. Thematic content and sub-themes described by traditional healers and utilizers of traditional medicine were combined in subsequent descriptions and were evaluated within the contexof our study goals. This study specifically focused on the reoccurring theme of mental health conditions from the perspective of Liberian traditional healers, and utilizers of traditional medicine. Sub-themes described by study participants that were associated with mental health conditions included culture-bound syndromes such as Open Mole, African Science, and Epilepsy. Study participants also described contemporary mental illness such as depression, trauma, and substance use [TABLE 2 AND TABLE 3]. Additionally, we asked study participants about social factors and their connection to mental health conditions, as well as attitudes and beliefs about mental health training and Western medicine.

**Table 1:** Participant Demographics

	N(%)	Mean Age (Years) [SD]
**Traditional Healers (N =** **24)**		**52.3 (13.6)** *
Male	15 (62.5)	53.5 (14.3)*
Female	9 (37.5)	47.8 (11.6)*
**Traditional Healer** **Identification**		
Bone Specialist	2 (8.3)	50.5 (13.4)
Herbalist	19 (79.2)	50.8 (14.4)*
Healer	2 (8.3)	60 (5.6)
Technician	1 (4.2)	60
**Utilizer of Traditional** **Medicine (N = 11)**		**32.3 (9.9)**
Male	6 (54.5)	24.5 (1.9)
Female	5 (45.5)	40.0 (8.6)

* Does not include unreported age data from 6 Traditional Healers.

**Table 2:** Summary of commonly shared sub-themes held by traditional healers stratified by gender

Gender (N)	Common Mental Health Problems	Problem Attribution	Treatment Rendered
Male (15)	Open Mole African Science Epilepsy Depression/Suicidal Ideation Mental Illness Socioeconomic Distress	**Open Mole:** tension, anemia, connection between head and heart, dehydration, heavy loads. Leads to abnormal behavior **African** **Science:** “throwing” curses **Epilepsy:** different types of epilepsy, some types are “genetic”, and other types are caused by evil spirits, African Science – throwing curses **Depression/Suicidal** **Ideation:** Loneliness and lack of social support, stress, poverty, unemployment **Mental** **illness:** war/trauma, substance use, demons and spirits	**Open Mole:** Herbal remedies **Epilepsy:** Herbal remedies **Depression:** Counseling **Mental Illness:** Herbal remedies, medication – antipsychotic (chlorpromazine) and benzodiazepine (diazepam)
Female (9)	Open Mole African Science “Craziness” Depression Socioeconomic Distress	**Open Mole:** small worms, pregnancy, heavy load, cardiovascular disease, “mental problems” **Mental** **Illness:** chronic open mole, stress, trauma **Trauma:** nightmares, spirits	**Open Mole:** Herbal remedies **Depression:** Counseling **African** **Science:** Herbal remedies **Epilepsy:** Herbal remedies

**Table 3:** Summary of commonly shared sub-themes held by traditional medicine utilizers stratified by gender

Gender (N)	Common Reasons for Seeing a Traditional Healer	Problem Attributions	Treatments Received
Male (6)	Inadequate medical services Cultural Preference Cost Cultural Idioms – Open Mole, African Science, Epilepsy - spirits	**Depression:** lack of hope, stress **Open** **Mole:** different from mental illness **Epilepsy:** not caused by African Science	**Open Mole:** Both traditional medicine and modern medicine **Epilepsy:** Believe that both traditional medicine and modern medicine can help
Female (5)	Lack of medical facilities Traditional beliefs	**Open** **Mole:** Carrying heavy loads **Mental** **illness:** frustration, stress, trauma, war **African** **Science:** witchcraft **Epilepsy:** genetic, biologic causes	**Open Mole:** Herbal remedies **Depression:** Counseling **Epilepsy:** Modern medication

### Open Mole

Open Mole was described as a sunken fontanel or depression in the head and is a cultural idiom of distress that has been commonly described among different ethnic groups in rural Liberian communities. When asked about common problems by researchers, study participants described Open Mole as a common problem in Liberia and provided narrative descriptions that included both physical and psychological symptoms. Study participants also described a connection between the “heart and the head”, which appeared to describe more of the psychosomatic features of this condition rather than actual cardiovascular disease, and dehydration.

Other symptoms attributed to Open Mole included somatic presentations – ‘depressed fontanel’, sinking or soft spot on head, migraine, pale, anorexia, anemia, generalized weakness, painful joints, and “heart pain”. Behavioral manifestations of Open Mole included “break downs”, inconsistent abnormal behaviors, “crazy”, hallucinations, worrying, irritability, infant fussiness, and speaking loudly. Below is a description of Open Mole and its connection to ‘heart disease’ by a 61-year-old male who identified himself as a traditional herbalist. The herbalist described what he believes causes Open Mole, how he treats Open Mole, and how his approach contrasts with ‘modern doctors’.

“*Ok it is the open mole which is heart disease...and that heart disease is connected to the, I will say connected to the whole entire vital organs and then the mole becomes depressed and then hole come through the mole that is the head the top of the head and our people sometimes used what you call it certain mud and mix with certain medicine.*”

“*What we have in our own traditional thinking is the nerves... the nerves control the body. Ok and so what we believe is that is going by the wave of the mind that is to say the heart wave. We have certain medicine that you will not force it but as soon as you take the heart wave will travel with the ah speed of the heart.*”

“*Well, to defend myself look at the reality well not everybody the same but ah they are sometimes good somewhere. Because we don't treat the symptoms of the disease we treat the disease*.”

A 24-year-old utilizer of traditional medicine described abnormal and unusual behaviors that someone with Open Mole might exhibit.

“*Open mole, it's like somebody behaving funny, at times the person behaves abnormal, refer to the person to be having open mole, the person is abnormal, yes. The person at times behaves funny; the actions, maybe you see somebody just stand maybe try to take off their shirt, take off their trousers, they can be having open mole*.”

Attributed etiologies of Open Mole ranged from African Science (curses), violating traditional beliefs, trauma, “tension” or stress, an infectious disease, results of the war, drugs, dehydration, “carrying heavy loads”, and high blood pressure. Below is a description of beliefs about Open Mole and its putative etiology from a 43-year-old male traditional healer.

“*Thank you very much, most of the thing that causes this open mole sometimes it comes from high blood pressure. Some come from dehydrate, when the person is dehydrate. So, when we I diagnose this person, when I diagnose I will know the pressure has caused the open mole. So how do I know? When I diagnose, after I have know the, I have checking the blood pressure I will check the head. Then if my hand, if this hand go in softly to the head here, I know you have open mole*.”

A female utilizer of traditional medicine (unknown age) stated that she was diagnosed with Open Mole by a medical doctor. She believed that the illness was due to “loading a heavy load”, and went on to state

“*I don't know if open mole is caused by African sign. I have rheumatism. I feel pain all over my body and I think it is because of the open mole I have*.”

Treatments for Open Mole varied, depending on how severely affected the patient appeared to be, but often involved the application of herbal remedies in some capacity. Below a 28-year-old male “herbalist” described what he learned about Open Mole, and how to treat it from his mother. That he learned traditional medicine from his mother was worth noting, as traditional medicine is typically passed from father to son or mother to daughter.

“*Ahn, now for mental illness the only healing for traditional healing that I understand from my mother is certain thing they call open mole*.”

“*There is a certain plant that we used in combination with another thing, we actually burn it, and we mixed it with oil and some medication. Firstly, you have to put it to the person's skull, the same something you burn another type that you used to the person nose*.”

### African Science

African Science, sometimes also referred to as African Sign in the participant narrative, were cultural idioms of distress used to refer to clusters of conditions described by study participants as an externally imposed physical or mental illness typically levied by “throwing sickness” onto someone mediated through curses, poisons in food, witchcraft, spells, or spiritual forces. One 56-year-old female traditional healer likened the physical appearance of a condition attributed to African Science to that of “ring worm” spreading up through the body. Societal or cultural motivations to throw curses or use witchcraft may be to cause embarrassment or pain to someone because the rights of others or traditional beliefs have been violated in some way. Other described conditions or symptomassociated with African Science varied widely and included open mole, “insanity or craziness”, hydrocele, pain all over the body, epilepsy, and hallucinations. A 68-year-old male traditional healer described psychosomatic symptoms along with cultural motivations, and connection with nature associated with African Science below. The connection between the moon and mental illness has been well-documented and seems to be incorporated in the cultural idiom of African Science^23^.

“*You see the sickness walk in the person, walk all in the body, it get in the head, from the head it walk in all then, sometimes you see the person weak, they don't have power. African Science. The throw sickness on the person. When you get the sickness, it always embarrass you, your living, it embarrass your life, sometimes it in your body, you sick every day, you can't get well. After sometimes, if it stops coming out now, if it stop throwing the person down now, then sometimes you watch the new, when the new moon comes up, then you put the medicine there*.”

### Epilepsy

“Epilepsy” was another cultural idiom of distress regularly described in study participant narratives. Epilepsy could be associated along with African Science but was also sometimes viewed as being independent of this condition. Different “types” of epilepsy were often associated with witchcraft, spiritual, or demonic possession. It was difficult to determine whether participant narrative represented emic descriptions of neurologic illness (i.e. recurring seizures), or if “epilepsy” referred to a different idiomatic phenomenon. Treatments for epilepsy consisted of herbal preparations with “special kinds of honey”. Some patients with epilepsy were also observed to have “kidney problems”. A male traditional healer (age unknown) described different types of epilepsy and referred to at least one type of epilepsy that he believed was due to spiritual forces.

“*And the third one [type of epilepsy] like you will always like you, its demonic I swear. Like you want to take bath, you stand somewhere you remember that somebody already talk to you to do something. Because I can interview people when they come to me so how it starts and this and that. And that treatment also depends on the symptoms*.”

“*Because if they get up they will you know there is something I also learned about epilepsy and I try to share that people don't believe it. All epileptic patients they have kidney problems...you know that?*”

### Depression/sadness

Depression or referred to interchangeably as “sadness” was a “common” condition often described as an entity separate from other forms of mental illness by study participants. Depression appeared to be more connected with one's set of extrinsic environmental circumstances, such as loss of a loved one, displacement, trauma from past conflict, poverty, social isolation, etc., rather than being viewed as a condition brought on by one's choices, such as substance use, or being afflicted with African Science, demonic possession, or the result of intrinsic biological pathology associated with mental illness.

A commonly described scenario among study participants was that family played an important role in providing psychosocial support by counseling their mentally ill relative, especially those who are depressed and suicidal. Pharmacologic treatment was sometimes used to calm anxiety or agitation, and to facilitate effectiveness of counseling, which was the primary treatment modality described among study participants. Below, a 68-year-old male traditional healer described how he helped a young mother who was distraught after losing her twin children using a combination of medication and counseling.

“*For the recent case, ah, the girl lost her twins, that was the most recent one and she almost wanted to commit suicide. She just wanted to end her life because she felt that she lost whatever she was suffering for. Well, we have some sedatives, diazepam and Vitamin B complex, we give her sedatives, and some other relatives came in to counsel her that all was not finish, she was still young, and she would still have children. We talk to the, the partner who also came in to give more assistance and assure her that he won't abandoned her, he will still be with her and all will work out. She's ok now, we give her to home, we give her some ah some other small, small sedative drugs that the mother manage. She, we didn't give it to her to taker herself, we give to the mother that at evening time give this, because she could go and take all to just end herself.*”

A 45-year-old female utilizer of traditional medicine described 'loneliness' and lack of guidance or friendship as a common cause of depression and sadness.

“*Maybe apparently there's no guidance, you know, like comforter, sometime when you are lonely, you have no advisor.*”

Other Mental Illness: “Frustration” and “Craziness”

Other mental illnesses were also alternatively referred to as “frustration”, and sometimes “craziness”, and were viewed as being separate from other conditions such as depression/sadness or Open Mole. Many of the beliefs about mental illness and the tradecraft treatment approaches were passed down generationally. Attributed etiologies of mental illness included witchcraft, drugs and alcohol, stress, trauma related to war, African Science, and demonic or evil spirit possession. Symptoms included hallucinations, unusual behaviors, and aggression. Such individuals could be segregated from the local population, and in some cases chained in isolation. Herbal remedies and sometimes antipsychotic or benzodiazepine medications were given in an effort to calm the person or facilitate removal of an evil spirit, and a period of abstinence from alcohol or other substances was often prescribed. Below, a 38-year-old male traditional healer described some of the challenges associated with substance use in addition to describing mental illnesses using the general term ‘frustration’, as well as the belief that at least some kinds of mental illness are a result of demonic possession.

“*Arum ah, for Africa, um, when we talk about mental illness we have about three to four types, kinds of mental illness. First we have um, those who take the narcotics, those who drink alcohol and those that are frustrated. Then in Africa we have something that we call the dark world people [referencing African Sign] sometimes they look at you in the family, or look at your family, and they ah, block your mind. And ah those who are demonically possessed*.”

A 23-year-old male utilizer of traditional medicine described the impact that war had on young people leading to trauma and substance use.

“*One of the major things that cause the problem is because the war have great side effect of the mind of the young people. Because for example during the war that's when many teenagers learn how to smoke, take in different types of drugs and because of that it has great impact on the brain*.”

### Social Determinants and Mental Health

Social determinants of mental health were not only described as secondary impediments to receiving quality mental health care but were themselves collectively viewed as a fundamental cause of mental health conditions and cultural idioms of distress that Liberians face. Unemployment, poverty, lack of education, nepotism, “disunity”, home and food insecurity, lack of facilities to care for marginalized persons (elderly, those with disability, those with mental illness, etc.), and lack of access to modern mental health care, were recurring, interrelated socioeconomic factors described by nearly all of the study participants. Below is a description from a 43-year-old male traditional healer.

“*The major problem in mental health in Liberia is that there is no center for mental health problems. There are no places for referral. Other problems are old age - people are abandoned. There is lack of support and poor finances. Also, lost members of family, lost worth, loss of jobs and political positions and loss of homes*.”

Poverty was cited by a female traditional healer who did not give her age as both a contributor to mental health problems, and as a barrier to receiving modern healthcare, making traditional medicine a more affordable alternative.

“*Yes, for family to cope with the situation that's they don't have enough money. And during the war many homes have been burned down to the......and then some lost members, supportive members so all this causing the problems of mental health and then...*”

“*Some of the reason again is because some people cannot afford to go to the hospitals actually. And African medicines and herbalists are not really expensive*.”

### Attitudes and Beliefs about Mental Health Training and Western Medicine

Attitudes and beliefs regarding mental illness varied, with some study participants expressing a desire for formal, standardized training for mental health conditions, recognizing the significant shortage of mental health services. A 79-year-old male traditional healer expressed a desire for more training and education for traditional healers to treat mental health conditions as well as a center for treating mental health conditions.

“*So I would want us to have paramedical psychiatric program to see high level we can start with before people pushing up at the university. Yeah, ah I am suggesting about a workshop wherein you will have people to attend and brief them almost looking like awareness so that they can know that there is a program in the area for mental health. You see. There is nobody even there are not nurses trained in psychiatric or psychiatric nursing they may be taught in classroom, but it had not been effective practically n*o.”

“*Ok for Liberia really the major problem in the health, let's look at the mental is that we do not have a center as a referral center for handling mental health conditions*.”

A male traditional healer (age unknown) described limitations in providing healthcare and when they would desire to leverage Western medicine providers to help provide care while still holding traditional medicine in high regard.

“*Well, ah, I have not done that the only thing I can do if you sick I do not understand the...in fact if I understand the symptom. I do not like to treat on assumption. I always want to get scientific proof because the thing herbalist cannot do is to take a particle from somebody a specimen and put it in the machine and tell exactly what happen. That's what western drugs I mean western tradition ah can do that. And ah they cannot open up to take something from inside and close. This two thing herbalists have not gotten to that. But actually when it comes to herbal medicine they can treat the whole person. You know what I mean?*”

A 28-year-old male traditional healers described frustration that many Western doctors did not want to collaborate with traditional healers.

“*The major problems for me I see the herbalist really function out of the city and then the other practitioner that is western practitioner, some of them or may be majority does not like to affiliate with us. They do not like to affiliate with us. Yes, like for instance for me, am an herbalist of more than one medicine, but mainly I cure sores. And then, when you want to maybe numb this place in some cases it critical, so we need some western practitioner to and you know help you to numb so that you can do the traditional dressing and healing*.”

Study participants also cited a strong preference for traditional medicine among their patients and indicated that traditional medicine was still an important part of their culture. Belief in God and spirituality were also important aspects of healthcare, and the belief that one someone could be healed from their ailment. A 35-yearold female utilizer of traditional medicine described the importance of tradition as well as being suspicious of modern medicine as being influential in her desire to continue to seek out traditional medicine over modern medicine.

“*Some reasons I will say that some of us we are highly traditional, and we don't believe in medication or let's say tablets and things. We believe highly in our traditional certain, so if we are confronted with some problems, we rather going down to the traditional side than to come up to the medical side*.”

A 23-year-old male utilizer of traditional medicine described fear of Western medicine as a common reason that Liberians turned to traditional medicine.

“*Really let me just give you just maybe one reason, like for our setting Africa, especially Liberia most people they're afraid of western medicine especially when it comes to injection and maybe the dizziness of drugs, so they prefer going to the traditional medicine, which of course it does not have any side effect.*”

Other utilizers of traditional medicine indicated their strong preference for going to see a medical doctor instead of a traditional healer. A 25-year-old male who had previously used traditional medicine described how his preference changed to go to the clinic or hospital rather than seeing a ‘country doctor’ – referring to a traditional healer.

“*If I sick now, because me it like when you some where you got adapt, you got to adapt yourself to the system where you are living, now if I get sick I will go straight to the clinic or the hospital. I wouldn't even think about country doctor because right now I don't believe in country doctor.*”

## Discussion

The purpose of this study was to learn more about the attitudes and beliefs that Liberian traditional healers and utilizers of traditional medicine have about mental health conditions, the causes of mental illness, accepted treatments, social determinants of mental health conditions, and Western medicine. This is especially relevant in a post-conflict country such as Liberia that has been left with endemic illiteracy, poverty, corruption, substance abuse and poor health care since the end of its second civil war in 2003^1^,^2^.

Key findings of this qualitative research project were that 1.) traditional healers and utilizers of traditional medicine described mental health conditions, social factors, and access to healthcare as some of the most important problems facing Liberia from their perspective. A previous analysis of Liberia's National Health Policy that described a high prevalence of mental health disorders in Liberia and called out substantial access-to-healthcare gaps, particularly in rural parts of the country, aligned with traditional healer and utilizers of traditional medicine narrative descriptions^24^. 2.) Traditional healers and utilizers of traditional medicine from this study also described the interconnected relationship between mental health, physical health, and social factors in their narrative descriptions of cultural idioms of distress and mental health conditions. This finding is validated by previous studies showing that persons suffering from mental illness in conflict-affected areas, such as Liberia, are more at risk for violence, suicidality, poor physical health and substance abuse^2^,^3^,^24^,^25^. 3.) Traditional healers and utilizers of traditional medicine were ambivalent in their attitudes towards Westernized medicine. This has important ramifications as more African countries seek to integrate etic and emic approaches to mental healthcare^12^,^26^. 4.) Lastly, social factors including poverty, lack of education, unemployment, and social isolation were described at some level by nearly all of the study participants as a fundamental cause or contributor to mental health conditions that were described in this study^27^.

Descriptions of culture bound syndromes and treatment approaches by traditional healers and utilizers of traditional medicine for conditions such as Open Mole, African Science, and epilepsy were consistent with what has previously been described in the literature^29^,^30^,^31^,^32^. A common thread linking these cultural idioms of distress were the manifestation of physical and psychological symptoms that were intertwined with local cultural and religious beliefs about these conditions^31^,^33^. Based on findings from this study it would seem that an understanding of local culture and beliefs as well as social context would be just as important as a modern understanding of physical and mental health conditions when treating a person suffering from Open Mole, epilepsy, or African Science^14^,^34^. Likewise, beliefs about depression/sadness and other types of mental illness such as trauma, substance use, and psychosis as well as described treatment approaches that included counseling, use of herbal remedies, and rudimentary use of benzodiazepine and antipsychotic medication were also consistent with previous findings^17^,^31^.

Traditional healers supported by scientific methods, tools and guidelines could make a significant contribution to improving access to mental health care particularly in rural Liberian communities^14^,^17^. Indeed, traditional healers in this study expressed a desire to work more closely with their Western medicine colleagues, but also reported feeling that this was not reciprocal. The lack of reciprocity to collaborate on the part of practitioners of modern medicine may be rooted in historical attitudes and beliefs towards traditional healers^17^.

Safety concerns around beliefs and treatment approaches held and practiced by traditional healers continue to be important considerations^17^. Western medical practitioners in other studies have reported that they are not in favor of referring patients to traditional healers and advise discontinuation of treatments prescribed by traditional healers^34^, as the efficacy of traditional treatment practices have not been formally established^16^,^35^. Furthermore, some western medical practitioners have expressed concern about potential human rights abuses from traditional healers, including the restraint and exploitation of patients^35^. Some traditional healers have also expressed disinterest in working with Western medical practitioners because of past-perceived ill treatment from western medical professionals or due to the fear that their methods will be exploited^34^. Lastly, much of the research on perceived benefits of integrating traditional healers with Western medical practitioners was conducted based on physical ailments, and not mental health issues. Thus, our findings could serve as an impetus for further research on the benefits of integrating traditional healers with modern approaches to mental health care in Liberia and similar countries.

Traditional healers and utilizers of traditional medicine in this study, particularly those that represented younger generations, reported more awareness of their limitations, benefits of modern medicine, and desired more formal structured training, which could mitigate safety concerns that have been raised in previous studies^17^. As a new generation of traditional healers emerges, a different approach becomes possible if a willingness to challenge past assumptions and work towards a mutual trust and respect between traditional healers and Western providers exists^26^. As an example of this effort, other African countries have developed policies to cultivate a safe environment for traditional healers and Western practitioners to collaborate in. Recognizing the high utilization and importance of traditional healing, South Africa passed the “Traditional Health Practitioners Act. No 35 of 2004.” This act serves to mainstream and standardize traditional health practices. A Traditional Healers Council of South Africa was created to provide registration and training of traditional healers, to further protect communities who utilize the services of traditional health practitioners^18^. Given that traditional healers are an important part of mental illness treatment in African populations, South Africa has also begun to integrate community nurses and traditional healers. Studies have shown a positive response from communities and respondents to this new shift in mental healthcare^26^.

Consumers of care in other African countries preferred concurrent treatment from traditional healers and western medical practitioners for medical conditions such as hypertension^36^. Consumers of medical care held beliefs that western and traditional medicine can complement one another^36^. Furthermore, older studies have demonstrated that traditional healers and western medical practitioners were being equally helpful to the patient^37^. A recent qualitative study conducted by authors from this research group found that collaboration among Liberian traditional healers and western medical practitioners continued to be common^38^. Additionally, it was found that traditional healers referred individuals to western medical practitioners for diseases that resulted in severe complications or when diagnostic testing, such as lab assessments, were thought to be essential for treatment^38^. Western medical practitioners were also reported to refer individuals to traditional healers when an illness was chronic and available western treatment options had failed, which often occurred when a patient presented with a serious mental illness^38^.

All available evidence indicates that traditional medicine is here to stay, and that the time to act on the mental health treatment gap in low- and middle-income countries is now^35^,^39^. The continent of Africa is experiencing a renewed interest in traditional medicine, and up to 80% of Africans seeking heath care incorporate traditional medicine as a significant component of their care^40^,^41^. The use of traditional healers is higher for persons suffering from mental illness compared with the general population, whereby traditional healers often serve as the first point of contact for such persons^1^,^2^. A growing number of sub-Saharan African countries are taking steps to integrate traditional medicine into their national health systems^19^,^42^,^43^. As more African countries move to adopt national policies and legal frameworks for integrating traditional health care practice with modern healthcare in a safe and effective manner, a continued understanding of traditional medicine, traditional healers, and what mental healthcare looks like from their perspective will be critical^17^. Although the study design and the findings from this study were insufficient to contribute to more robust discussion, sustained improvement in population mental health seems unlikely to be achieved without addressing social factors through public policy and systemic intervention^28^. This would be an important consideration for further research.

## Limitations

This study sought build on the growing body of research surrounding the role of the traditional healers in healthcare within developing countries – in this case Liberia, and specifically mental healthcare by using an in-depth, semi-structured interview process to gather views, opinions, and narrative directly from traditional healers, and utilizers of traditional medicine. Such information is invaluable in informing larger qualitative and quantitative epidemiologic studies, and research questions. However, due to the relatively small sample size, and subjective nature of the narrative response, care must be taken when attempting to form generalized conclusions or extrapolate the data in other settings with different that must be considered. Selection bias and how this color the narrative should be considered. The authors strived to recruit a participant sample that would be representative of the different counties of Liberia. However, over a quarter of the population is in the city of Monrovia and surrounding townships, making it difficult to recruit from more rural areas of Liberia. This study also focused on two perspectives - that of the traditional healer, and utilizers of traditional medicine. We did not gather narrative from other healthcare or mental healthcare providers to compare differing perspectives on the nature of mental healthcare and potential role for traditional healers in Liberia. This would be an important research question for future study.

## Conclusions

Given the prominent role that traditional medicine plays in the treatment of mental health conditions, and the growing utilization of traditional medicine in Liberia and other African countries with similar profiles, our hope is that the findings from this study will contribute to a greater knowledge about traditional medicine in Liberia, an understanding of opportunities and barriers to the utilization of mental health care, and future intervention efforts such as integrating modern mental healthcare with traditional medicine.

## Declarations

All supporting data used in the production of this manuscript is kept on file at the Boston University School of Medicine, Department of Psychiatry, Boston Medical Center campus, Boston, MA. You may contact either the corresponding author (first author) for any questions regarding the access of this data set. Their contact information is listed on the Title Page.
